# NLDB: a database for 3D protein–ligand interactions in enzymatic reactions

**DOI:** 10.1007/s10969-016-9206-0

**Published:** 2016-08-16

**Authors:** Yoichi Murakami, Satoshi Omori, Kengo Kinoshita

**Affiliations:** 1 Graduate School of Information Sciences, Tohoku University, Aramaki-Aza-Aoba 6-3-09, Aoba-ku, Sendai, 980-8575 Japan; 2 Institute of Development, Aging and Cancer, Tohoku University, Seiryo-machi 4-1, Aoba-ku, Sendai, 980-8575 Japan; 3 Tohoku Medical Megabank Organization, Seiryo-machi 2-1, Aoba-ku, Sendai, 980-8573 Japan; 4 Center for Drug Design Research, National Institutes of Biomedical Innovation, Health and Nutrition, Saito-Asagi 7-6-8, Ibaraki-city, Osaka, 567-0085 Japan

**Keywords:** Protein–ligand interactions, Docking, Enzymatic reactions, Variant residues, Database

## Abstract

NLDB (Natural Ligand DataBase; URL: http://nldb.hgc.jp) is a database of automatically collected and predicted 3D protein–ligand interactions for the enzymatic reactions of metabolic pathways registered in KEGG. Structural information about these reactions is important for studying the molecular functions of enzymes, however a large number of the 3D interactions are still unknown. Therefore, in order to complement such missing information, we predicted protein–ligand complex structures, and constructed a database of the 3D interactions in reactions. NLDB provides three different types of data resources; the *natural* complexes are experimentally determined protein–ligand complex structures in PDB, the *analog* complexes are predicted based on known protein structures in a complex with a similar ligand, and the ab initio complexes are predicted by docking simulations. In addition, NLDB shows the known polymorphisms found in human genome on protein structures. The database has a flexible search function based on various types of keywords, and an enrichment analysis function based on a set of KEGG compound IDs. NLDB will be a valuable resource for experimental biologists studying protein–ligand interactions in specific reactions, and for theoretical researchers wishing to undertake more precise simulations of interactions.

## Introduction

Protein–ligand interactions play key roles in almost all biological processes, ranging from enzyme catalysis to signal transduction. The molecular recognition of a ligand by its host protein requires non-covalent interactions, such as hydrogen and hydrophobic bonding, between molecules. Thus, characteristics of these interactions, including the ligand-binding modes and binding affinities, are valuable information to facilitate the elucidation of molecular mechanisms of ligand recognition to understand molecular functions in vivo.

The large-scale structural information for protein–ligand interactions is currently available in the Protein Data Bank (PDB; [2]), and has contributed to the physicochemical analyses of their interactions. However, this wealth of information is not still enough to physicochemically explain all of the enzymatic reactions with the enzymes that are important potential drug targets [6].

The information about various metabolic pathways and their related reactions has been manually curated and stored in the Kyoto Encyclopedia of Genes and Genomes (KEGG; [7]). In the KEGG REACTION database, enzymes that catalyze reactions are linked to their structural information in PDB, if their structures are known and accessible. However, even if the structure of an enzyme catalyzing a reaction of interest is available in PDB, its structures in a complex with substrates or products in the reaction are not always experimentally determined. In such cases, detailed information about the 3D interaction characteristics in natural ligands may not be obtained.

Due to the importance of the information about 3D protein–ligand interactions for studying the molecular functions of enzymes, it would be valuable to organize and complement the missing structural information of the metabolic pathways with computational approaches. There are some high-quality databases of biologically relevant ligands bound to proteins, such as Binding MOAD [1] and BioLiP [22], however, these databases have not registered such missing structural data, which can be pre-calculated with predictions. Therefore, we collected and predicted the complex structures of protein–ligand interactions by focusing on the interactions in the reactions of the KEGG REACTION database, and then constructed the database, named NLDB (capitals denote Natural Ligand DataBase; URL: http://nldb.hgc.jp).

**Fig. 1:**
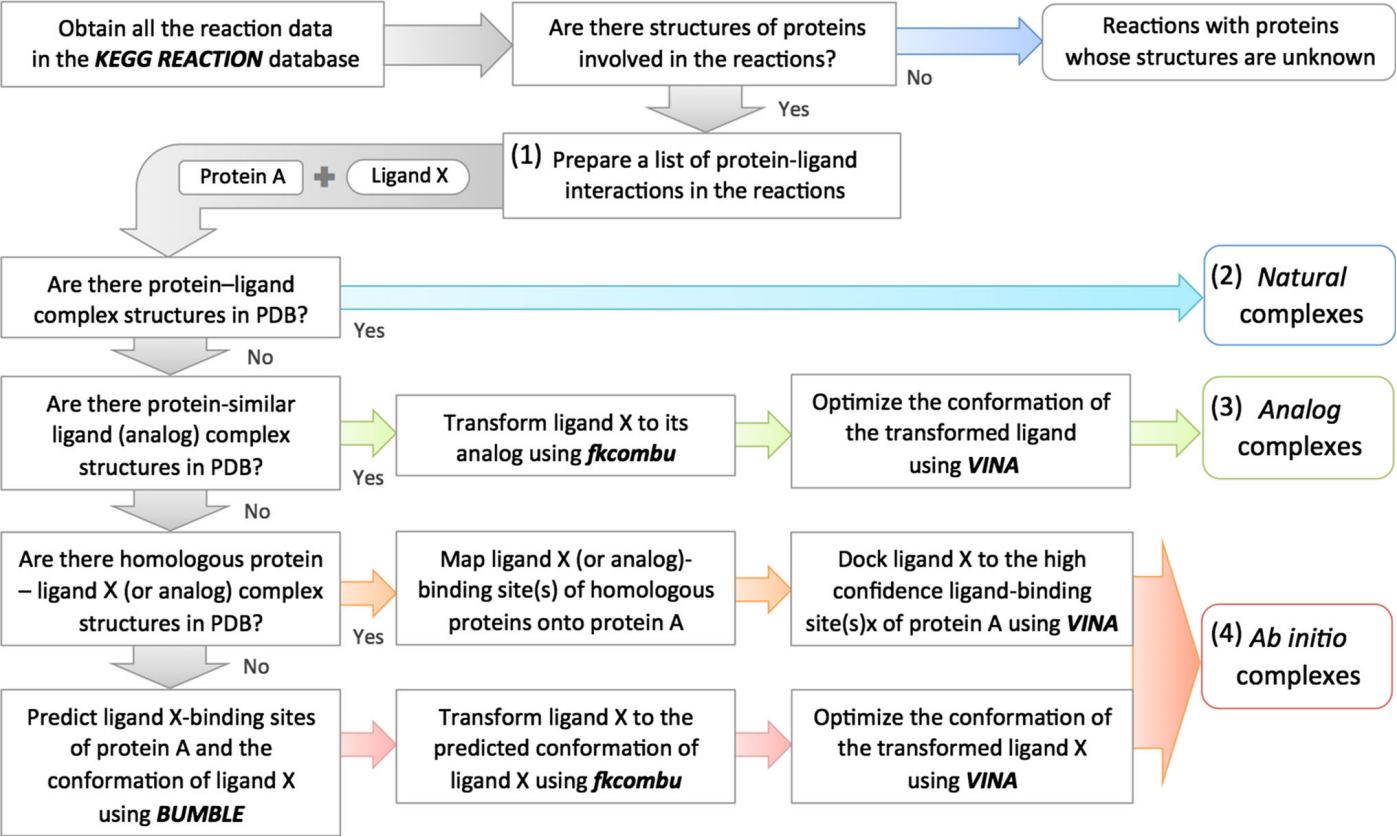
Schematic diagram of the NLDB data construction

NLDB deals with the compounds observed in the KEGG reactions, and provides three different types of data resources: *natural*, *analog*, and ab initio complexes (Fig. 1; see “Materials and methods” for details). NLDB provides a flexible search function, based on various types of relevant keywords, and an enrichment analysis function based on a set of KEGG compound IDs. NLDB is a unique, up-to-date database that not only collects 3D protein–ligand interactions from known structures but also automatically predicts complex structures that were previously unknown.

## Materials and methods

### Procedure for the NDLB data construction

The NLDB data were constructed through four main steps: (1) preparation of a list of protein–ligand interactions in enzymatic reactions (2) collection of *natural* complex structures, (3) prediction of *analog* complex structures, and (4) prediction of ab initio complex structures (Fig. 1).

#### Preparation of a list of protein–ligand interactions in enzymatic reactions

All the reactions in the KEGG REACTION database along with the KEGG IDs for the metabolic pathways, enzymes and compounds related to each reaction were obtained using the KEGG API [7, 8]. In KEGG, the reactions are manually curated, and each reaction has information of reactants (substrates and products). In addition, other IDs corresponding to each of the enzymes, such as PDB IDs and UniProt accession numbers, and chemical compound IDs found in the PDB chemical component dictionary (PDB-CCD; [21]) for ligands bound to the enzyme, were also obtained using the same API. Then, these IDs were merged together, and reactions with enzymes to which PDB IDs were assigned, i.e. whose structures were known from the KEGG REACTION database, were selected for the list for the NLDB data construction. On the other hand, the other reactions with enzymes to which PDB IDs were not assigned were not included in the list, and were immediately registered in NLDB.

The following two clustering processes were then carried out. The first clustering for a set of chemical compounds was done by the chemical similarity scores (Tanimoto coefficients) between two different compounds, calculated by the connected maximum common substructure (C-MCS) search using the *fkcombu* program [10]. In C-MCS, the substructures between two small molecules are defined as a connected graph, and have the same atom types and bond connections. The Tanimoto coefficient of 0.7 was used as the threshold to define the similar compound groups, because the average RMSD of 3D conformations was reportedly 2.0 Å for compound pairs with more than 0.7 chemical similarity [11]. The second clustering for a set of proteins was done by sequence similarity using the CD-HIT program [13] with the default sequence identity threshold of 0.9, and with the option of 0.8 for the alignment coverage of a longer sequence. In each cluster, protein sequences were further aligned using the *clustalw2* program [12], and then locations about their known ligand-binding residues within 4.5 Å from any atoms of a ligand were shared and mapped between the aligned sequences.

The two data sets were then merged together, to form three different types of ligand–protein complex lists: (I) a list of known protein–ligand complex structures, (II) a list of known protein-analog complex structures, and (III) a list of known *apo*-protein structures. To make the list (III), proteins with a ratio of missing residues (#missing residues/#total residues in the protein) of more than 10 % were removed from the list (III), and a representative protein structure with the largest sequence length, which precedes structures with the highest resolution, was selected for each reaction on the same list.

#### Collection of *natural* complex structures

The *natural* complexes were defined as the complex experimentally determined and registered in PDB. We used the word ‘*natural*’ to distinguish compounds naturally found in vivo from compounds artificially generated in vitro. Note that NLDB deals with only the compounds found in the KEGG REACTION database. According to the list (I), the coordinates of the ligands in complex with proteins were extracted from the PDB files.

#### Prediction of *analog* complex structures

**Fig. 2:**
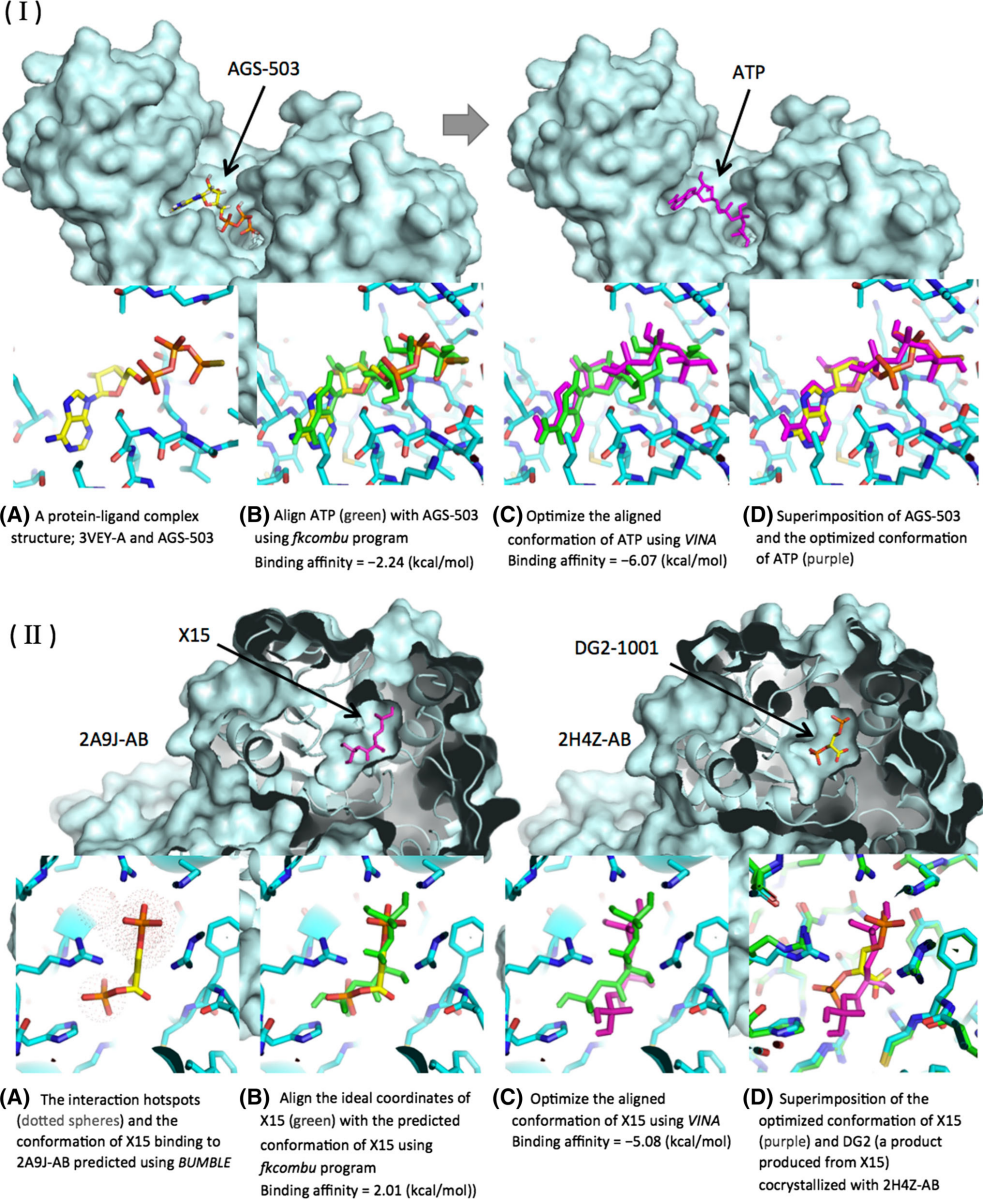
Procedure for predictions of *analog* and ab initio complexes. (**I**) A complex structure of 3VEY-A and ATP (adenosine 5′-triphosphate; C_10_H_16_N_5_O_13_P_3_) in KEGG reaction: R00299 is predicted, based on the complex structure of 3VEY-A and AGS-503 (Phosphothiophosphoric acid-adenylate ester; C_10_H_16_N_5_O_12_P_3_S). The chemical similarity score calculated based on C-MCS is 0.94. **A** The complex structure of 3VEY-A and AGS-503 is used as the template for the construction of a new 3VEY-A and ATP complex structure. **B** An ATP (*green*) is aligned with AGS-503, using the *fkcombu* program. The aligned conformation of ATP has the binding affinity (VINA docking score) of −2.24 (kcal/mol). **C** The aligned conformation of ATP (*purple*) is optimized using VINA, with the local optimization option. The optimized conformation of ATP has the binding affinity of −6.07 (kcal/mol). In addition, the optimized conformation of ATP superimposed onto AGS-503 is shown in **D**, for reference only. (**II**) An ab initio complex structure of bisphosphoglycerate mutase (PDB ID: 2A9 J-AB) and X15 (1.3-Bisphosphoglyceric acid; C3 H8 O10 P2) in KEGG reaction: R01662 is predicted, according to the following steps: **A** Interaction hotspots are predicted, and then the energetically favorable conformations of X15 in the predicted hot spots are also predicted, using BUMBLE. **B** The ideal coordinates of X15 (*green*) are aligned with the predicted conformation, using the *fkcombu* program. The aligned conformation of X15 has the binding affinity of 2.01 (kcal/mol). **C** The aligned conformation of X15 (*purple*) is optimized using VINA, with the local optimization option. The optimized conformation of X15 has the binding affinity of −5.08 (kcal/mol). **D** The superimposition of the optimized conformation of X15 onto its product, DG2 (2.3-Diphosphoglyceric acid; C3 H8 O10 P2), cocrystallized with the same protein (PDB ID: 4H4Z-AB), is shown for reference only

The *analog* complexes were protein–ligand complex structures, predicted based on the structures of protein-analog interactions according to the list (II). Firstly, the ideal coordinates of compounds, computed with the CORINA program, which automatically generates high-quality and low-energy 3D coordinates for a small molecule [17], were downloaded from the Ligand Expo database [4]. Secondly, a target compound known to bind a target protein in a reaction was superimposed onto its analog bound to the protein, using the *fkcombu* program, which flexibly transforms a target molecule onto a reference molecule and is bundled in the KCOMBU package program for comparison and modelling of chemical structures [10, 11] (Fig. 2I-B). Finally, the conformation of the superimposed compound was locally optimized using AutoDock VINA [18], which is a fast molecular docking program that can be used for large virtual screening (Fig. 2I-C). In addition, the program calculated the binding affinity (VINA docking score) for each binding conformation (pose), and the poses with lower affinities were considered to be more probable.

#### Prediction of ab initio complex structures

The ab initio complexes are protein–ligand complex structures predicted based on the docking simulation, according to the list (III). The quality for ab initio complexes is largely dependent on the accuracy of the predictions of the binding sites and the initial conformations of ligands. Thus, we used two different flows to construct complex structures. When homolog information about the ligand-binding sites was available, the target compound was docked onto the binding sites on the target protein using VINA. When homolog information was not available, the target-specific ligand-binding sites and the energetically favorable conformations of a target ligand were predicted using BUMBLE, which predicts them based on known fragment–fragment interactions observed in PDB [9] (Fig. 2II-A). BUMBLE reported that the average success rate of the conformations with the RMSD of <5.0 Å between the native and predicted ligands was 53 % for the first-ranked-predicted conformations and 70 % in the top 10 conformations in the test for bound structure [9]. However, even though the prediction of the ligand-binding sites was reportedly more accurate than AutoDock [14], the conformations of the compounds built from the predicted interaction hotspots were still less accurate [9]. Therefore, the ideal coordinates of the target compound were superimposed onto the predicted conformation using the *fkcombu* program, and then the aligned conformation was optimized using VINA, in the same manner as the data construction of the *analog* complexes (Fig. 2II-B, C).

### Keyword search and enrichment analysis

**Fig. 3:**
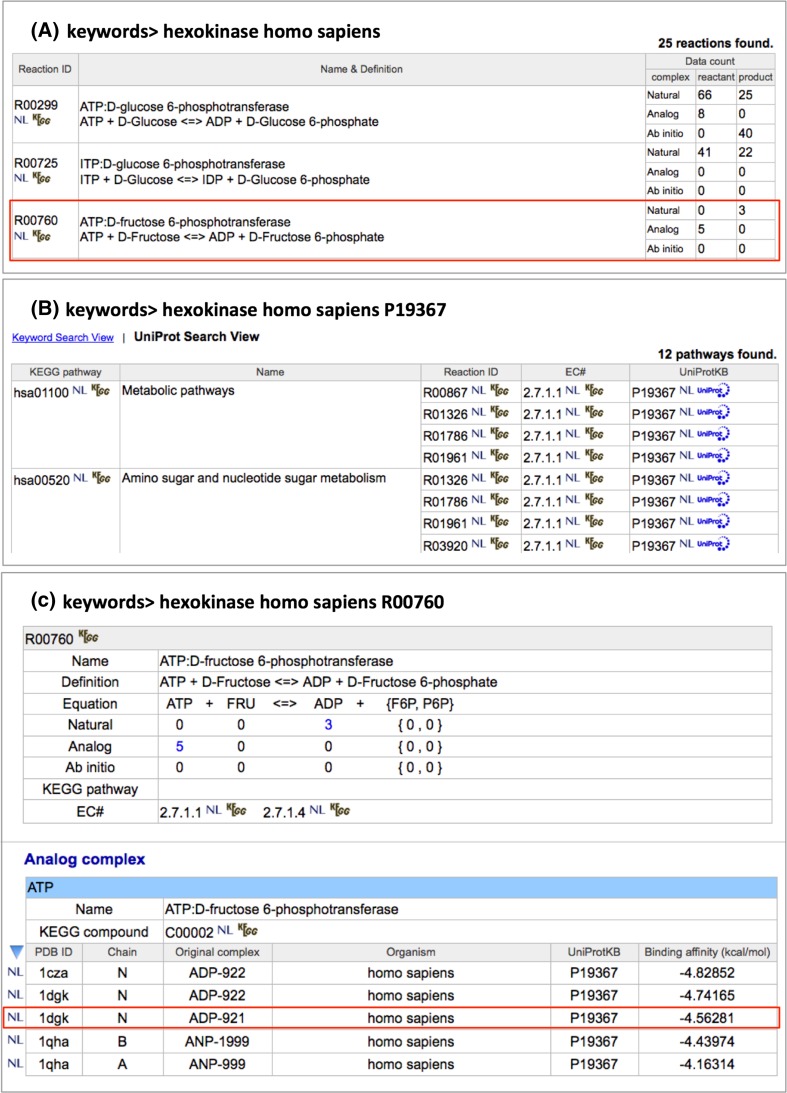
Examples of a keyword search in NDLB. **A** When a set of keywords, for example, ‘*hexokinase, homo sapiens’* is inputted in the search box of the NLDB top page, a list of the reactions with the data counts of three types of complex structures, *natural*, *analog,* and ab initio is obtained. **B** In addition, in the case when at least one UniProt AC number (e.g. ‘*P19367’*) is also inputted in the search box, a list of KEGG pathways associated with their reactions is displayed by switching it to the ‘*UniProt Search View*’. **C** Then, lists of the available complex structures for the reaction selected in the result page A or B, e.g. ‘*R00760’* in the row surrounded by a red square on page A, are obtained. The different types of structures in complex with a substrate and a product are shown in different tables. In addition, when the input keywords include a KEGG reaction ID or when only a KEGG reaction ID is inputted, then page B will be directly accessed, without going through page A

**Fig. 4:**
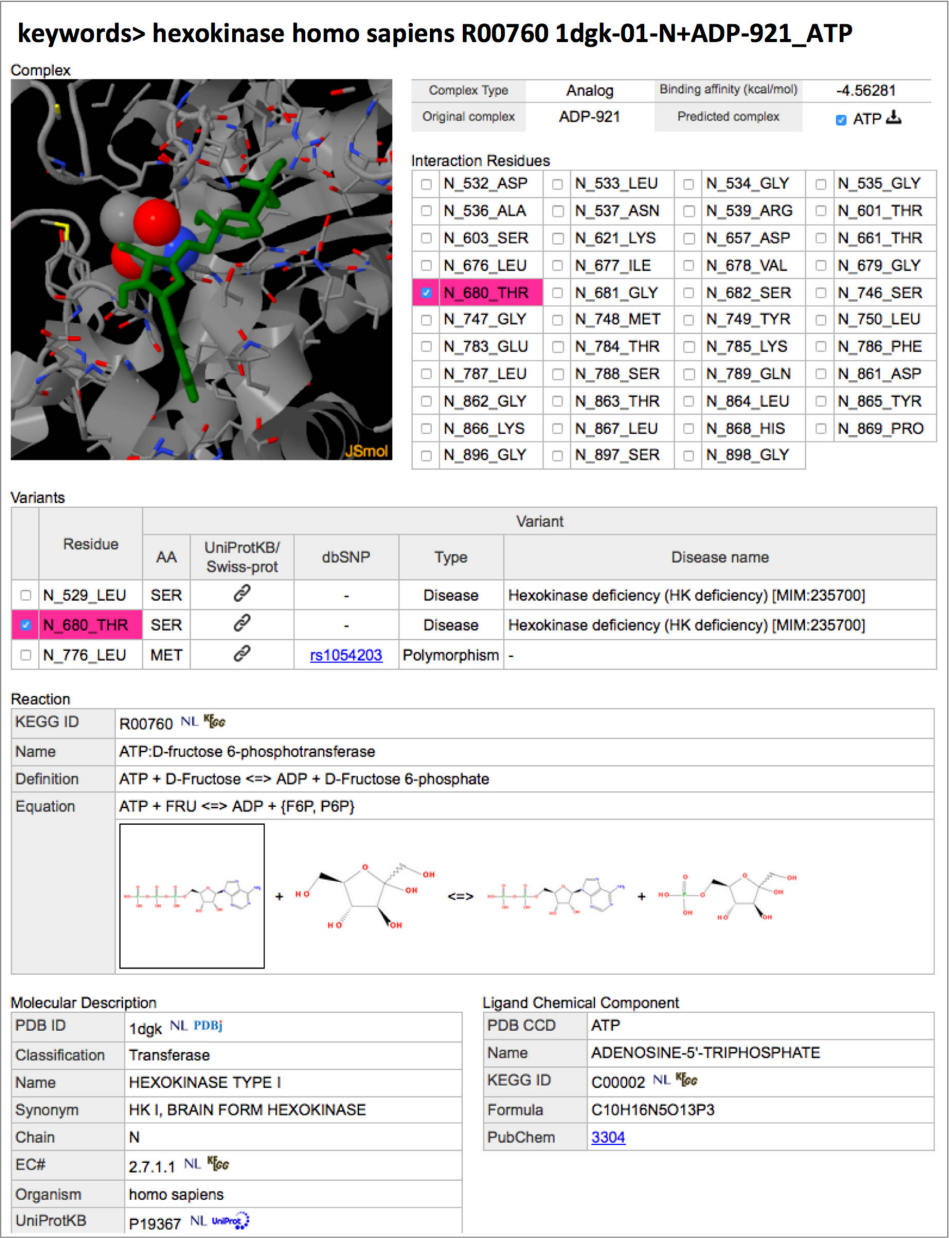
An example of the search results page of a protein–ligand complex structure. A list of interacting residues within 5.0 Å from the ligand atoms is shown in the ‘*Interaction Residues*’ table, and the complex structure is also visually displayed in the JSmol panel, in the top left-hand corner. For human proteins, variants including human polymorphisms and disease-associated mutations are also shown in the ‘*Variants*’ table, and are highlighted if there are variants in the binding site of the protein. In this example of the *analog* complex of 1DGK-N and ATP, a variant residue, 680-THR in chain N, located in the binding site is specified in the table of interaction residues (*highlighted in pink*). Other detailed information about the reaction, the molecule, and the compound is also shown and linked to the external databases, such as KEGG, PDBj and UniProt

NLDB provides a flexible keyword search function, enabling users to retrieve the structures of particular protein–ligand interactions in reactions of interest. Various types of keywords are allowed in the search function, such as *PDB entry ID*, *molecule name*, *organism*, *compound name*, *KEGG reaction ID*, *EC number*, *UniProt accession number*, *chemical component ID, rs number, OMIM ID* and so on, as well as combinations of these keywords. In this function, partial matching is selected by default, so that, for example, the keyword of ‘*GMP*’ matches not only a chemical ID but also protein names. Perfect matching can be achieved as an option, in this case, for example, the keyword of ‘*GMP*’ matches only a chemical ID. According to the keywords inputted in the search box of the NLDB top page, the following three result pages will be provided: (1) The first result page is a list of reactions with the data counts of three types of complexes, *natural*, *analog,* and ab initio (Fig. 3A). In addition, in the case when at least one UniProt AC number is submitted as a keyword, a list of KEGG pathways associated with their reactions is displayed by switching it to the ‘*UniProt Search View*’ (Fig. 3B). (2) The second page is a list of available complex structures for a particular reaction of interest submitted as a keyword or selected in the first page (Fig. 3C). The data counts of protein structures with a specific ligand, substrate or product in each complex type are shown in the top table, and the lists of different types of complex structures are also shown, in different tables. The binding affinities for each *analog* or ab initio complex structure are also shown in the Table. (3) The third page shows the detailed information of a specific protein–ligand complex structure (Fig. 4). On this page, a list of interacting residues within 5.0 Å from the ligand atoms is shown in the ‘*Interaction Residues*’ table, and the complex structure is also visually displayed in the JSmol panel, in the top left-hand corner. For human proteins, variants including human polymorphisms and disease-associated mutations are also shown in the ‘*Variants*’ table, and are highlighted if there are variants in the binding site of the protein. The data set of variants with sequence positions and variation types, polymorphisms or disease associate mutations, is collected in *humsavar.txt*, which is an index of manually curated variants from UniProtKB/Swiss-Prot, and downloaded from UniProt [19]. Other detailed information about the reaction, the molecule, and the compound is also shown and linked to the external databases, such as KEGG, PDBj and UniProt.

NLDB also provides an enrichment analysis of a set of KEGG compounds. This function enables users to retrieve enriched KEGG pathways with expected *p*-values. In addition, a list of the reactions in each enriched pathway is also shown, in the same table format as that in the first result page of the keyword search.

### An example: complementation of missing complex structures

NLDB can be used to complement the missing complex structures in chemical reactions as in the following example.

Bisphosphoglycerate mutase (BPGM; EC: 5.4.2.4) is an erythrocyte-specific enzyme, and its main function is to regulate the oxygen affinity of hemoglobin by controlling the synthesis of 2.3-bisphosphoglycerate (DG2; C3 H8 O10 P2), which is an allosteric effector of hemoglobin, via a phosphoryl transfer reaction [15, 20]. The deficiency of BPGM (BPGMD) increases the hemoglobin oxygen affinity, leading to a decrease in the DG2 concentration, and is characterized by hemolytic anemia [3]. There are three relevant reactions catalyzed by BPGM in the KEGG REACTION database, and they can be searched with the keywords ‘*bisphosphoglycerate mutase*’ in NLDB (KEGG reaction IDs; R01516, R01518 and R01662). In particular, BPGM in the complex with 1,3-bisphosphoglycerate (X15; C3 H8 O10 P2) in R01662, which is the main function of BPGM, to convert X15 to DG2, is unknown in PDB. This missing complex structure would be strongly required, for clarifying the binding mode of the ligand and also for identifying key residues in the reaction. In the case of BPGM with X15, there are no *analog* complexes in which a ligand similar to X15 binds. In addition, the chemical similarity score between X15 and DG2, based on C-MCS, is 0.43. Thus, the ab initio complex structure was predicted, according to our data construction procedure (Fig. 2II). The final conformation of X15 bound to BPGM (PDB ID: 2A9 J-AB) is shown in Fig. 2II-D. Note that water atoms are not considered in the docking calculation, even though water molecules can enhance ligand stability and activity, by forming hydrogen bonds or water bridges. As a consequence, X15 was predicted to have a docking pose on the same binding pocket as DG2, with a low binding affinity of −5.08 (kcal/mol) (Fig. 2II-C, D). Furthermore, it was observed that X15 forms two hydrogen bonds with one of the variant residues of BPGMD, ARG-62, as well as a hydrogen bond with CYS-23, which is considered to have a large effect on the reactivity of BPGM [16]. Moreover, ARG-90, which is a variant with a large effect on the stability of the protein [5], participated in the binding. While this ab initio complex structure of BPGM and X15 is reasonable, in terms of the binding affinity and the binding mode, it represents a starting point for further detailed analyses to evaluate the stability of the predicted pose, considering the effects of both the protein flexibility and water solvation.

## Discussion and conclusions

NLDB is a unique, up-to-date database that collects 3D protein–ligand interactions from known structures, and also automatically predicts missing complex structures using reliable, state-of-the-art software programs, in the reactions of the KEGG REACTION database. As far as we know, there are no comparable databases focused on protein–ligand complex structures involved in these reactions.

NLDB registers 68,551 *natural*, 28,441 *analog* and 64,204 ab initio complexes, for 3248 KEGG reactions in which 1654 enzymes are involved, and also registers 4379 KEGG reactions in which 3291 enzymes without structural information are involved (As of July 2016). In total, 7627 reactions have been registered in NLDB. Furthermore, 1679 and 2131 entries with variant residues in their binding sites linked to rs number and OMIM ID, respectively, are registered and viewed in the ‘*Variants*’ page. The former entries are associated with 89 pathways and 367 reactions, and the latter entries are associated with 103 pathways and 354 reactions.

Even though experimentally determined complex structures; i.e., *natural* complexes, cover only 19.09 % (1456/7627) and 21.66 % (1652/7627) of all registered reactions, which have at least one known protein structure in a complex with a substrate and a product, respectively, the current coverage of 3D protein–ligand interactions in NLDB is 35.23 % (2687/7627) and 21.19 % (2885/7627) of these reactions, which have at least one known or predicted protein structure in a complex with a substrate and a product, respectively (Table 1). These numbers were obtained by checking whether at least one known or predicted protein structure in a complex with a substrate or a product exists in each reaction. In addition, NLDB can provide the predicted structures of protein–ligand interactions with binding affinities of ≤−3.0 for about 22 % of the registered reactions, and those with binding affinities of ≤−5.0 for about 13 % of the registered reactions.

**Table 1:** The number of the reactions with 3D protein–ligand interactions registered in NLDB

Complex type	#Substrate (%)	#Product (%)	#Substrate (%)	#Product (%)	#Substrate (%)	#Product (%)
			Binding affinity ≤−3.0		Binding affinity ≤−5.0	
*Natural*	1456 (19.09)	1652 (21.66)	–	–	–	–
*Analog*	793 (10.40)	822 (10.78)	698 (9.15)	686 (8.99)	556 (7.29)	550 (7.21)
*Ab initio* ^*1^	60 (0.79)	57 (0.75)	53 (0.69)	53 (0.69)	47 (0.62)	36 (0.47)
*Ab initio* ^*2^	1333 (17.54)	1469 (19.26)	1120 (14.68)	1196 (15.68)	461 (6.04)	443 (5.81)
All	2687 (35.23)	2885 (37.83)	1616 (21.19)	1688 (22.13)	959 (12.57)	934 (12.25)

The number (#) of reactions, which have at least one known or predicted protein structure in a complex with a substrate or a product, was counted for each complex type. In addition, the number of reactions, which have at least one predicted complex structure with binding affinity (VINA docking score) of ≤−3.0 or −5.0, was counted for each complex type. The number in parentheses shows the percentage of the reactions with structural information in all of the reactions registered in NLDB, 7627 reactions including 4379 reactions without structural information (As of July 2016). *Ab initio* ^*1^ and *Ab initio* ^*2^ correspond to complex structures predicted based on high confidence or predicted information of ligand-binding sites, respectively (see the section ‘Construction of ab initio complexes’)

In particular, we believe that these predicted structures with lower binding affinity can provide some insights for experimental biologists studying protein–ligand interactions in specific chemical reactions, in which the 3D structures of the interaction are as yet unknown, and will facilitate breakthroughs in understanding or verifying chemical reactivities at specific ligand-binding sites, as shown in the above example. Furthermore, NLDB will be a starting point for theoretical researchers wishing to undertake more accurate simulations of the ligand-binding affinity and stability in the predicted conformation of a complex, by considering the effects of protein flexibility and water solvation. Therefore, NLDB will be continually improved, for the prediction of more accurate structures involved in reactions and for web interface usability. NLDB is freely accessible at http://nldb.hgc.jp, and will be regularly updated every 3 months.

## References

[1] Ahmed A, Smith RD, Clark JJ, Dunbar JJ, Carlson HA (2015). Recent improvements to Binding MOAD: a resource for protein–ligand binding affinities and structures. Nucleic Acids Res.

[2] Berman H, Henrick K, Nakamura H (2003). Announcing the worldwide Protein Data Bank. Nat Struct Biol.

[3] Bowdler AJ, Prankerd TA (1964). Studies in congenital non-spherocytic haemolytic Anaemias with specific enzyme defects. Acta Haematol.

[4] Feng Z, Chen L, Maddula H, Akcan O, Oughtred R, Berman HM, Westbrook J (2004). Ligand Depot: a data warehouse for ligands bound to macromolecules. Bioinformatics.

[5] Garel MC, Lemarchandel V, Calvin MC, Arous N, Craescu CT, Prehu MO, Rosa J, Rosa R (1993). Amino acid residues involved in the catalytic site of human erythrocyte bisphosphoglycerate mutase. Functional consequences of substitutions of His10, His187 and Arg89. Eur J Biochem.

[6] Imming P, Sinning C, Meyer A (2006). Drugs, their targets and the nature and number of drug targets. Nat Rev Drug Discov.

[7] Kanehisa M, Goto S (2000). KEGG: kyoto encyclopedia of genes and genomes. Nucleic Acids Res.

[8] Kanehisa M, Goto S, Sato Y, Furumichi M, Tanabe M (2012). KEGG for integration and interpretation of large-scale molecular data sets. Nucleic Acids Res.

[9] Kasahara K, Kinoshita K, Takagi T (2010). Ligand-binding site prediction of proteins based on known fragment-fragment interactions. Bioinformatics.

[10] Kawabata T (2011). Build-up algorithm for atomic correspondence between chemical structures. J Chem Inf Model.

[11] Kawabata T, Nakamura H (2014). 3D flexible alignment using 2D maximum common substructure: dependence of prediction accuracy on target-reference chemical similarity. J Chem Inf Model.

[12] Larkin MA, Blackshields G, Brown NP, Chenna R, McGettigan PA, McWilliam H, Valentin F, Wallace IM, Wilm A, Lopez R, Thompson JD, Gibson TJ, Higgins DG (2007). Clustal W and Clustal X version 2.0. Bioinformatics.

[13] Li W, Godzik A (2006). Cd-hit: a fast program for clustering and comparing large sets of protein or nucleotide sequences. Bioinformatics.

[14] Morris GM, Huey R, Lindstrom W, Sanner MF, Belew RK, Goodsell DS, Olson AJ (2009). AutoDock4 and AutoDockTools4: automated docking with selective receptor flexibility. J Comput Chem.

[15] Patterson A, Price NC, Nairn J (2010). Unliganded structure of human bisphosphoglycerate mutase reveals side-chain movements induced by ligand binding. Acta Crystallogr, Sect F: Struct Biol Cryst Commun.

[16] Ravel P, Craescu CT, Arous N, Rosa J, Garel MC (1997). Critical role of human bisphosphoglycerate mutase Cys22 in the phosphatase activator-binding site. J Biol Chem.

[17] Sadowski J, Gasteiger J, Klebe G (1994). Comparison of automatic three-dimensional model builders using 639 X-ray structures. J Chem Inf Comput Sci.

[18] Trott O, Olson AJ (2010). AutoDock Vina: improving the speed and accuracy of docking with a new scoring function, efficient optimization, and multithreading. J Comput Chem.

[19] UniProt C (2015). UniProt: a hub for protein information. Nucleic Acids Res.

[20] Wang Y, Liu L, Wei Z, Cheng Z, Lin Y, Gong W (2006). Seeing the process of histidine phosphorylation in human bisphosphoglycerate mutase. J Biol Chem.

[21] Westbrook JD, Shao C, Feng Z, Zhuravleva M, Velankar S, Young J (2015). The chemical component dictionary: complete descriptions of constituent molecules in experimentally determined 3D macromolecules in the Protein Data Bank. Bioinformatics.

[22] Yang J, Roy A, Zhang Y (2013). BioLiP: a semi-manually curated database for biologically relevant ligand-protein interactions. Nucleic Acids Res.

